# A stable two-component cationic liposome platform for mRNA delivery induces CD8^+^ T-cell responses and protection in a murine lymphoma model

**DOI:** 10.1186/s12951-026-04234-3

**Published:** 2026-03-07

**Authors:** Gabriel Kristian Pedersen, Reham Sabah Alhakeem, Ahmad Tami, Dennis Christensen, Zahra Shabanian, Rune Fledelius Jensen, Katharina Wørzner, Signe Tandrup Schmidt

**Affiliations:** 1 Department of Infectious Disease Immunology, Center for Vaccine Research, Statens Serum Institut, Artillerivej 5, Copenhagen, 2300 Denmark; 2 Department of Immunology and Microbiology, Faculty of Health and Medical Sciences, University of Copenhagen, Copenhagen, Denmark; 3 Vaccine Adjuvant Systems R&D, Croda Pharma, Lyngby, 2800 Denmark

**Keywords:** Cationic liposome, MRNA, Cancer vaccine, Cytotoxic T cell

## Abstract

The mRNA-LNP-based SARS-CoV-2 vaccines were a breakthrough for the technology; however, their production requires specialized equipment, and the complex formulation involving four distinct lipid classes imposes stringent requirements on both characterization and quality control. We tested a cationic adjuvant formulation containing a simple two-component liposome formulation based on dimethyldioctadecylammonium bromide and monomycoloyl glycerol-1 (CAF^®^04) for mRNA-delivery, which can be complexed with mRNA by simple admixing on-site. The physicochemical characteristics of the resulting vaccine particles depended on the CAF04:mRNA N/P ratio, where an N/P ratio ≤ 1.09 resulted in anionic particles with little aggregation and N/P ratios ≥ 2.18 resulted in increasingly cationic particles that aggregated. These attributes influenced the magnitude of the CD8^+^ T-cell responses induced against OVA-encoding mRNA and at optimal CAF04:mRNA N/P ratios (0.76–1.42), the antigen-specific CD8^+^ T-cell responses reached magnitudes comparable to the DOTMA:DOPE Lipoplex (RNA-LPX) platform. Therapeutic vaccination using CAF04:mRNA delayed tumor growth and enhanced survival in an E.G7-OVA lymphoma model. We present a highly stable mRNA delivery platform that can be freeze-dried for extended storage and admixed with mRNA on-site to induce highly functional CD8^+^ T-cell responses with potential for therapeutic cancer vaccines.

**Graphical Abstract:**

**Figure:**
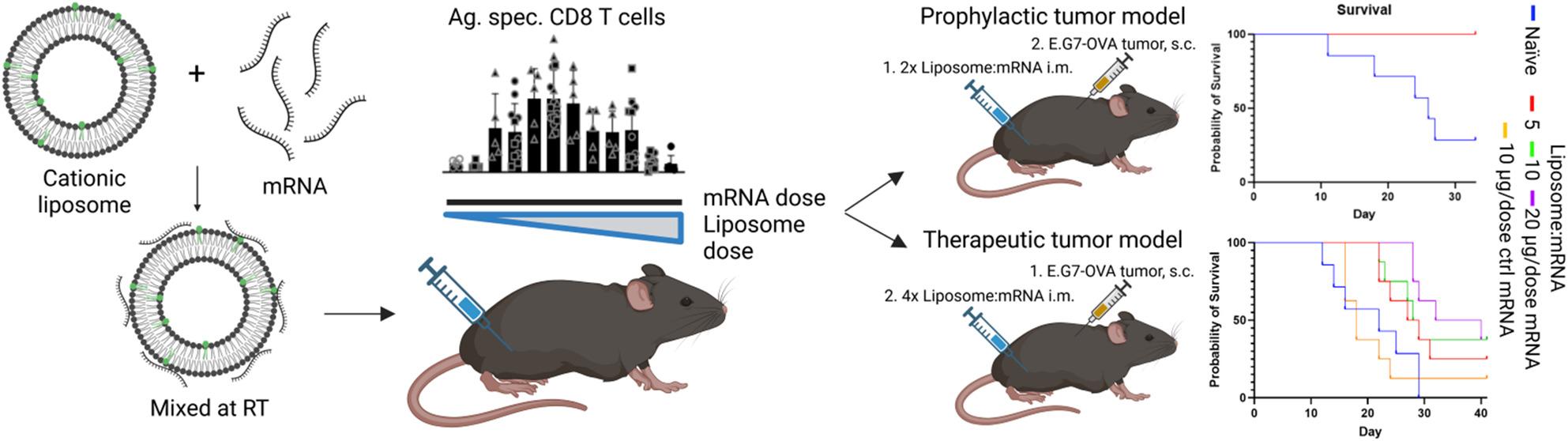
Created in BioRender. Pedersen, G. (2026) https://BioRender.com/4m7zipc

**Supplementary Information:**

The online version contains supplementary material available at 10.1186/s12951-026-04234-3.

## Introduction

Personalized vaccines hold promise as cancer therapies, utilizing somatic mutations in tumors as antigens in vaccines, with a number of clinical studies showing a positive effect on disease progression [1–3]. The somatic mutations may be sufficiently different from ‘self’ to act as epitopes for CD8^+^ T cells and are unique to the individual patients, i.e. neoantigens [4]. It follows that the neoantigens must be identified and manufactured as part of the treatment for each cancer patient, and can be prepared as either peptides or neoantigen-encoding mRNA or DNA [2, 5]. Manufacturing the neoantigens as peptides may pose difficulties, as they might not be easily synthesized due to their biophysical characteristics, and consequently may not be included in the final therapeutic vaccine [5]. In contrast, nucleotide-based vaccines, e.g. mRNA-based, will have essentially the same physicochemical properties independent of the sequence, and manufacture of personalized neoantigen-encoding mRNA will therefore be more reliable and quicker to GMP approve than using peptides, which could ultimately increase the speed of neoantigen-based vaccine production.

Vaccines comprising antigen-encoding mRNA complexed within lipid nanoparticles (LNPs) were tremendously successful in bringing a halt to detrimental societal effects of the COVID-19 pandemic. When formulated with LNPs, the mRNA is located inside the particles [6]. This process, effectively protects the mRNA from degradation and facilitates delivery into target cells, but requires specialized equipment and dedicated production facilities, which may hamper or slow down production of vaccines with mRNA encoding varying antigens, e.g. for personalized treatment of cancers. Another suitable strategy for personalized mRNA vaccine delivery is to apply complexation with a cationic delivery system. In this way, the mRNA may be simply admixed with pre-formulated delivery system for rapid vaccine production. The use of cationic polymers is well researched, and e.g. poly(ethyleneimine) (PEI) is routinely used for in vitro delivery of mRNA, while the natural polymer chitosan is explored for in vivo delivery of mRNA [7]. Complexation of mRNA with cationic liposomes is also being explored as a vaccination strategy. Cationic DOTMA:DOPE-liposomes forming lipoplexes with antigen-encoding mRNA (from here on defined as mRNA-LPX in accordance with original literature) induces cytotoxic CD8^+^ T-cell responses in both mice and humans after intravenous (i.v.) administration [8, 9]. In mice, the vaccine showed both prophylactic and therapeutic anti-tumor effects in the B16, B16F10, CT26 and TC-1 tumor models [8]. In a clinical trial using mRNA encoding several different tumor-associated antigens complexed with LPX-liposomes in a therapeutic regimen, polyclonal CD4^+^ and CD8^+^ T-cell responses were induced and several patients experienced partial remission of the disease [9, 10]. Using another approach, cationic liposomes based on dimethyldioctadecylammonium bromide (DDA) derivates formed lipoplexes with mRNA by mixing the lipids dissolved in ethanol, 96% (EtOH) and the mRNA in aqueous buffer by microfluidics [11]. Effective translation of mRNA and induction of antigen-specific antibody responses was observed in the lungs after i.v. injection of the mRNA-cationic liposome complexes, whereas modest expression was observed after intramuscular (i.m.) administration [11].

The cationic adjuvant formulation (CAF^®^) family is composed of liposomal formulations based on the cationic lipid DDA in combination with various immunostimulators with the purpose of tailoring the immune responses to different pathogens [12]. These include CAF01 (DDA stabilized with the Macrophage-inducible C-type lectin [Mincle] agonist trehalose-6´,6-debehenate, [TDB]) and CAF04, which also contains DDA in combination with the human Mincle agonist, synthetic monomycoloyl glycerol-1 (MMG-1) [13, 14]. It was previously demonstrated that complexation of self-amplifying RNA (saRNA) with CAF01 resulted in RNA translation at the site of injection and induction of CD4^+^ T-cell and antibody responses against a *Chlamydia Trachomatis* antigen [15]. Based on these encouraging results, we hypothesized that complexing antigen-encoding mRNA with CAF^®^-based adjuvants had the potential to induce tumor-specific CD8^+^ T-cell responses, required for effective cancer vaccines [8, 16]. We tested CAF04, as a highly stable two-component platform for mRNA delivery, which can be freeze-dried to ease distribution. Although CAF04 has not been in clinical trials, the adjuvant is expected to be safe in humans as the same lipids have been tested as part of the CAF09b adjuvant (additionally containing polyinosinic-polycytidylic acid [pIC]) in two phase I clinical studies for delivery of peptide antigens [17, 18]. The complexation between mRNA and CAF04 was performed by gentle pipette mixing, thus not requiring specialized equipment or personnel training for formulation and the method is therefore suitable for ‘bed side’ mixing at vaccination sites, e.g. for personalized therapeutic cancer vaccines at hospitals. For this project, we used mRNA encoding chicken egg ovalbumin (OVA) as a model antigen and investigated in detail the parameters required for optimal expression and immunogenicity. The vaccines were tested at different mRNA to liposome ratios and using various immunization routes. Furthermore, the functionality of induced antigen-specific CD8^+^ T-cell responses were evaluated in a prophylactic and therapeutic OVA-expressing E.G7 s.c. tumor model.

## Materials and methods

### Materials

DDA, MMG-1 and TDB were purchased from NCK A/S (Farum, Denmark). OVA-mRNA (5MoU) and eGFP-mRNA (5MoU) were acquired from TriLink BioTechnologies (San Diego, CA, USA), while Spike SARS-CoV-2 mRNA (5 MoU) was from Oz Biosciences (Marseille, France). All other chemicals were used at analytical grade and purchased from commercial suppliers.

*Preparation of liposomes*: Weighed amounts of DDA and MMG-1 were dissolved in EtOH and transferred to glass vials at a 5:1 DDA:MMG-1 weight ratio (82:18 molar ratio, selected based on the optimal ratio defined elsewhere [13]). Thin lipid films were formed by evaporating the EtOH under a gentle N_2_ stream followed by air-drying overnight. The lipid films were rehydrated in Tris-buffer (10 mM, pH 7.0, 2% w/v glycerol) to a final DDA: MMG-1 concentration of 2.5:0.5 mg/ml. The vials were placed on a water bath at 60 °C, and high-shear mixed by using a Heidolph Silent Crusher equipped with a 6 F shearing tool (Heidolph Instruments GmbH, Schwabach, Germany) at 26,000 rpm for 15 min. Glycerol is used in the buffer to stabilize the liposomes and tonicity. Fluorescently labelled CAF04 was prepared by adding 2% w/w 1,1’-Dioctadecyl-3,3,3’,3’-Tetramethylindodicarbocyanine, 4-Chlorobenzenesulfonate (DiD, Thermo Fisher Scientific, Waltham, MA, USA) to the lipid bilayer during formation of the thin lipid film. Liposomes based on DOTMA and DOPE (1:1 molar ratio, 1:1.1 mg/ml) were prepared using a similar high-shear mixing method in Tris-buffer (10 mM, pH 7.0, 2% w/v glycerol).

*Complexation of CAF- or DOTMA:DOPE-liposomes and mRNA*: mRNA and CAF- or DOTMA:DOPE-liposomes were diluted separately in RNase-free water and Tris-buffer (10 mM, pH 7.0, 2% w/v glycerol), respectively, to equal volumes. Then the diluted CAF- or DOTMA:DOPE-liposomes were added to the diluted mRNA while pipette mixing gently, and allowed to complex at room temperature for 15 min. prior to use. Several N/P ratios of CAF04:mRNA were prepared (Table 1), while DOTMA:DOPE-liposomes were complexed with mRNA at a 0.29 N/P-ratio, creating LPX:mRNA, according to Kranz et al. [8].

**Table 1:** Weight ratios and doses of CAF04:mRNA at N/P ratios used in in vivo studies. CAF04 doses were calculated based on an mRNA dose of 5 µg, and an average mRNA molar weight of 330 g/mol

*N*/*P* ratio	CAF04:mRNA weight ratio	DDA: MMG-1 dose (µg)
0.13	0.3	1.25:0.25
0.26	0.6	2.50:0.50
0.44	1.0	4.17:0.83
0.52	1.2	5.00:1.00
0.76	1.75	7.29:1.46
1.09	2.5	10.42:2.08
1.42	3.25	13.54:2.71
1.74	4.0	16.67:3.33
2.07	4.75	19.79:3.96
2.18	5.0	20.83:4.17
3.27	7.5	31.25:6.25
4.36	10.0	41.67:8.33

*Physicochemical characterization*: The average hydrodynamic particle sizes, polydispersity indexes and zeta potentials were measured by dynamic light scattering (DLS) using a Zetasizer Nano ZS (Malvern Instruments Ltd, Worcestershire, UK) equipped with a 633 nm laser and 173° detection optics. For particle size measurements, the samples were diluted 10x in mQ water, while the samples were diluted 100x in mQ water for measuring the zeta potentials. The level of adsorption of mRNA to CAF04 was qualitatively evaluated by running the CAF04:mRNA complexes in a 1% agarose gel (1 µg mRNA/well, 100 V, 40 min) using Sybr safe (Invitrogen, Carlsbad, CA, USA) as detection agent and visualized in a UV SynGene Bio Imaging system (SynGene, Cambridge, UK) using the GENESys software v.1.6.9.0.

### Cryogenic transmission electron microscopy (cryo-TEM)

Undiluted samples of uncomplexed CAF04 or complexed with OVA-mRNA at weight ratios 2.5 and 5 were imaged by cryo-TEM at the Core Facility for Integrated Microscopy at University of Copenhagen. The samples were placed on a Pelco lacey carbon grid, blotted and snap-frozen in liquid nitrogen. The images were acquired by using a Tecnai G2 20 TWIN transmission electron microscope (FEI, Hillsboro, OR, USA) mounted with a 4 × 4 K charged coupled device Eagle camera from FEI. An acceleration voltage of 120 kV and magnification of 29,000x was used for all samples.

*RNase A protection assay*: CAF04:eGFP-mRNA at N/P ratio 1.09 and 2.18 (0.05 µg/µl) were treated with 0.125 µg/ml RNase A (ThermoFisher Scientific) at RT for 30 min, followed by degradation of RNase A with 0.5 mg/ml protein kinase K (PK) at 50 °C for 15 min. Complexed mRNA was released by treatment with 1% Triton-X 100 (Tx-100, Sigma Aldrich) and 4 mg/ml heparin (hep, Sigma Aldrich) at 50 °C for 15 min. As control, samples were not treated with RNase A or Tx-100/hep, or neither. eGFP-mRNA was used as a positive control, while mRNA treated with RNase A was applied as negative control. The mRNA levels were assayed by gel electrophoresis as described above, though run for 20 min at 100 V.

*Heparin displacement assay*: CAF04:eGFP-mRNA complexes at N/P ratio 1.09 and 2.18, were incubated with heparin (Sigma-Aldrich) at 37 °C for 1 h. Concentration of mRNA: 0.025 mg/ml, heparin: 0, 0.125, 0.25, 0.5, 1, 2 and 4 mg/ml diluted in Tris-buffer, 10 mM, pH 7.0. Released mRNA was measured by using the Ribogreen assay (Thermo Fisher Scientific) according to the manufacturer’s instructions, and the ratio of release was calculated based on a pure mRNA control with 4 mg/ml heparin to account for potential interference.

*In vitro evaluation*: The transfection efficacy of CAF04:eGFP-mRNA complexes was evaluated in vitro in THP-1 cells (a kind gift from Camilla Foged lab, University of Copenhagen). The cells were maintained in RPMI 1640 (Invivogen) supplemented with 10% (v/v) heat-inactivated fetal calf serum, 5 × 10^− 6^ M β-mercaptoethanol, 1% (v/v) penicillin-streptomycin, 1% (v/v) sodium pyruvate, 1 mM L-glutamine, and 10 mM HEPES (cRPMI) at 37 °C and 5% CO_2_ and passaged at approx. 70% confluency. For the transfection studies, the cells were seeded at 10^5^ cells/well in 96-well tissue culture plates (Nunc, Hillerød, Denmark) in unsupplemented RPMI 1640. The mRNA was complexed with CAF04 at 3.0 µg/ml and diluted three times in RPMI 1640 prior to addition to the cells at 0.1 µg mRNA/well. Naked mRNA was used as a negative control, while linear PEI MW 40,000 (PEI MAX, Polysciences, Warrington, PA, USA) mixed at a 5:1 weight ratio with mRNA was used as a positive control. The cells were incubated with the mRNA complexes for 4 h at 37 °C, and subsequently pelleted by centrifugation at 500 *g* for 3 min. The supernatant was carefully removed from the wells not disturbing the cells, and replaced with warmed cRPMI while resuspending the cells. The cells were returned to the incubator for 40 h, and the eGFP expression evaluated by imaging on the ImageXpress Pico automated cell imaging system (Molecular Devices, San Jose, CA, USA) and by flow cytometry in the FITC channel on a Cytoflex (Beckman Coulter, Brea, CA, USA).

### In vivo evaluation

In all animal studies, C57BL/6JOlaHsd mice (Envigo, The Netherlands) aged 8–10 weeks at study start were used. All studies were conducted in accordance with European Council Directive 86/609/EEC and the Danish governmental Animal Experiments Inspectorate licenses 2017-15-0201–01363 and 2023-15-0201–01549. The size of study groups was determined by experience from previous studies or pilot studies, and studies were non-blinded and non-randomized. The mice were allowed to acclimatize for one week prior to commencing studies, and allowed free access to water, chow and environmental enrichment. Prior to commencing studies, a protocol stating hypotheses, experimental plan including measurements to alleviate pain and distress, and an analysis plan, was approved by the supervising veterinarian.

The immunogenic potential of CAF04:mRNA complexes was evaluated in female C57BL/6 mice (*n* = 4–6 mice/group depending on the experiment), which were immunized two times i.m. in the quadriceps, s.c. at the base of the tail or i.v. depending on the study with 1–25 µg mRNA/dose and CAF04 determined by weight ratio. At termination, the blood and spleens were harvested. The plasma was collected from the blood and the PBMCs were separated by using Lympholyte (Cedarlane, Burlington, CA, USA). The organs were processed to single cell suspensions using a nylon mesh cell strainer (Corning Inc., Corning, NY, USA).

The level of antigen-specific CD8^+^ T cells in blood and spleens was evaluated by multimer flow cytometry using the panel H2-K^b^-SIINFEKL:PE (ProImmune, Oxford, UK) and Fc block (BD Biosciences, San Jose, CA, USA), anti-mouse CD8:PerCP-Cy5.5 and CD4:APC-eFluor780 (both Thermo Fisher Scientific), and CD19:PECy7 and CD44:APC antibodies (both from BD Biosciences). Splenocytes were stimulated with the minimal CD8 epitope SIINFEKL, anti-CD28 and anti-CD49d in cRPMI for 1 h at 37 °C, brefeldin A was added and the cells were further stimulated at 37 °C for 5 h. The cells were treated with Fc block and surface stained with anti-mouse CD44:FITC, CD8:PerCp-Cy5.5 and CD4:APC-eFluor780 antibodies (all Thermo Fisher Scientific). Following permeabilization with Cytofix/Cytoperm (BD Biosciences), the cells were intracellularly stained with anti-mouse IFN-γ:PE-Cy7, TNF-α:PE and IL-2:APC antibodies (all Thermo Fisher Scientific). Antibody clones and dilution factors are indicated in Suppl. Table 1. All flow cytometry assays were acquired on a BD Fortessa flow cytometer and analyzed with FlowJo v10 (BD Biosciences). The total population of CD8^+^ T cells producing any of the cytokines, IFN-γ, TNF-α and IL-2, was calculated by using the OR gate in FlowJo, creating a population defined as all cells producing one, two or three cytokines.

### In vivo specific lysis

The cytotoxic potential of antigen-specific CD8^+^ T cells was evaluated by in vivo specific lysis. Single cell suspensions of splenocytes from naïve mice were divided in 6 equal portions in RPMI 1640 (Invivogen), and stained with 0.1, 1 or 10 µM CFSE and 2 or 20 µM CTV in all combinations by incubation for 10 min at 37 °C, followed by pulsing with 0, 0.0625, 1.25, 2.5, 5 and 10 µg/ml SIINFEKL peptide for 90 min at 37 °C inspired by Quah et al. [19]. Thus, the resulting pulsed cell populations were 0/0.1/20, 0.0625/0.1/2, 1.25/1/20, 2.5/1/2, 5/10/20 and 10/10/2 µg/ml SIINFEKL/µM CFSE/µM CTV. The cells were washed and counted, then the cells were pooled with equal amounts of live cells from each population in PBS (2 × 10^6^ cells/population, 12 × 10^6^ cells in total in 200 µl). The cells were transferred by i.v. injection in the tail vein to immunized mice and a naïve control group. At 24 h after administering the pulsed cells, the mice were euthanized and blood and spleens were collected and processed to single cell suspensions as described above, and the different cell populations were identified by using a Fortessa flow cytometer and analyzed with FlowJo v10 (BD Biosciences).

The percentage of specific lysis was calculated thus [20]:

$$\begin{aligned}\mathrm{S}\mathrm{p}\mathrm{e}\mathrm{c}\mathrm{i}\mathrm{f}\mathrm{i}\mathrm{c}\;\mathrm{l}\mathrm{y}\mathrm{s}\mathrm{i}\mathrm{s}\left(\%\right)& \quad=\left(1-\frac{{\left(\frac{pulsed}{unpulsed}\right)}_{immunized}}{{\left(\frac{pulsed}{unpulsed}\right)}_{na{i}ve\left(mean\right)}}\right)\quad\times100\%\end{aligned}$$

*Cancer studies*: In the prophylactic cancer study, female C57BL/6 were immunized twice i.m. in alternating quadriceps with 5 µg/dose OVA-mRNA complexed with CAF04 at N/P ratio 1.09 or LPX at N/P-ratio 0.29 at a 14-day interval. At 7 days after the final immunization, the mice were inoculated i.d. in the right flank with 5 × 10^5^ E.G7-OVA cells (CRL-2113, ATCC, Manassas, VA, USA) in 50 µl PBS, and the tumor growth was monitored daily. In the therapeutic cancer study, female C57BL/6 were inoculated i.d. in the right flank with 5 × 10^5^ E.G7-OVA cells (CRL-2113, ATCC) in 50 µl PBS. Subsequently, the mice were immunized four times, on day 4, 8, 13 and 18, i.m. in alternating quadriceps with 5, 10 or 20 µg/dose OVA-mRNA complexed with CAF04 at N/P ratio 1.09, with CAF04:Spike-mRNA (10 µg/dose) and Tris-buffer immunized groups as negative controls, and the tumor growth was monitored daily. The tumor volume was calculated using the modified ellipsoidal formula: V = 0.5xLxWxW [21], where L is the longest part of the tumor and W is perpendicular to L. A V = 800 mm^3^ was set as a humane end-point at which the mice were euthanized. Humane end-points were also developing an ulcer on the tumor at max. 5 mm in diameter or a weight loss of more than 20% (measured every 3 days). However, no mice met those end-points in the study. Mice that did not develop a visible s.c. tumor in the therapeutic study and the naïve control group of the prophylactic study were excluded due to ethical concerns, as the tumors might locate to the peritoneum. The area under curve (AUC) for tumor volume was calculated for individual mice. For mice euthanized during the study, the tumor volume was set to 800 mm^3^ for the remainder of the study from the day of euthanization [22].

### Statistics

The statistical difference between different treatment groups was evaluated by one-way analysis of variance (ANOVA) followed by Dunnett’s posttest or Tukey’s multiple comparisons test or Kruskal-Wallis test followed by Dunn’s posttest using GraphPad Prism v.10.1.2. (GraphPad Software LLC, La Jolla, CA, USA). Survival in the therapeutic tumor study was assessed by Log-rank (Mantel-Cox) test using GraphPad Prism v.10.1.2.

## Results and discussion

### Admixing of mRNA with CAF04 resulted in effective transfection of THP-1 cells

**Fig. 1:**
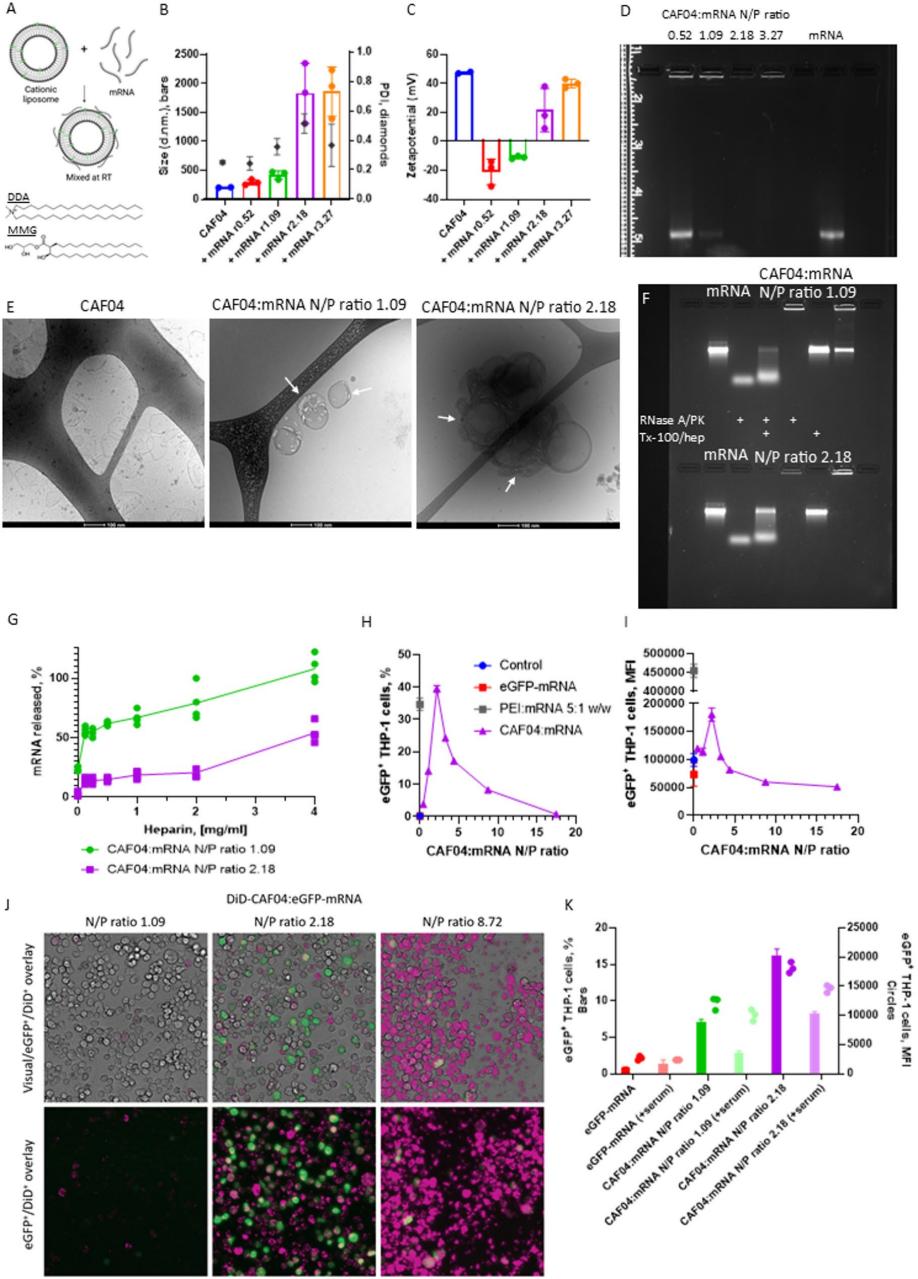
The N/P ratio affects resulting physicochemical characteristics and in vitro transfection efficacy. **(A)** Pre-prepared CAF04, composed of the lipids DDA and MMG-1, and OVA-mRNA were complexed by simple mixing by pipetting at different N/P ratios affecting **(B)** the particle sizes (weighted hydrodynamic diameter, bars) and polydispersity indexes (PDI, diamonds) and **(C)** the zeta potentials. *n* = 3, bars represent mean ± S.D. **(D)** The qualitative levels of uncomplexed OVA-mRNA was evaluated by agarose gel electrophoresis. **(E)** Cryo-TEM images of CAF04 complexed with OVA-mRNA at N/P ratios 1.09 and 2.18, or uncomplexed as a control. Electron beam damage was used as an indicator of mRNA presence (arrows). **(F)** mRNA degradation profile for CAF04:eGFP-mRNA at N/P ratio 1.09 (top row) and 2.18 (bottom row) following incubation with RNase A for 30 min at RT with subsequent incubation with protein kinase K (PK) at 50 °C for 15 min (denoted by + for RNase/PK). Remaining mRNA was released from the nanoparticle complexes by treatment with Triton-X 100 and heparin (Tx-100/hep denoted by +). Untreated samples and eGFP-mRNA were included as controls. **(G)** Heparin displacement of mRNA from CAF04:mRNA complexes. CAF04:eGFP-mRNA at N/P ratio 1.09 and 2.18 were treated with heparin at different concentrations at 37 °C for 1 h. The released eGFP-mRNA was measured by the Ribogreen assay and calculated as the ratio of eGFP-mRNA in 4 mg/ml heparin. *n* = 4. **(H)** The transfection efficiency was evaluated in undifferentiated THP-1 cells at different CAF04:eGFP-mRNA N/P ratios with 0.1 µg mRNA/10^5^ cells, the percentage and **(I)** mean fluorescence intensity (MFI) of eGFP expressing THP-1 cells was assayed by flow cytometry. **(J)** Qualitative assessment of eGFP expression and CAF04 adsorption at selected DiD-CAF04:eGFP-mRNA N/P ratios was performed by fluorescent microscopy. Green: eGFP, pink: DiD. **K)** Transfection of undifferentiated THP-1 cells with CAF04:eGFP-mRNA at N/P ratio 1.09 and 2.18 in cell media with and without 10% FCS. Transfection efficacy in % eGFP^+^ cells (left y-axis, bars) and MFI (right y-axis, dots). *n* = 2–3 technical replicates, the studies were repeated twice. A) Was created in BioRender: Pedersen, G. (2025) https://BioRender.com/rf1avcz

Formulation of mRNA with CAF04 was facilitated by simple admixing of the components by pipetting (Fig. 1A). Due to the electrostatic interactions of the anionic mRNA and the cationic liposomes, complexation is hypothesized to be virtually instantaneous. Admixing of CAF04 and mRNA at low N/P ratios (≤ 1.09) resulted in modest increases in particle size, as measured by Dynamic light scattering (DLS) and the particles were highly anionic (Fig. 1B, C). In contrast, high N/P ratios (≥ 2.18) resulted in an overall cationic surface charge and caused aggregation of the particles, indicated by an increase in the PDIs. Complementing these results, presence of free mRNA was assessed by agarose gel electrophoresis. In accordance with the apparent surface charge of the CAF04:mRNA-particles, free mRNA was observed at N/P ratios ≤ 1.09, while at higher N/P ratios all mRNA was complexed with CAF04 (Fig. 1D, Suppl. Figure 1). The CAF04:mRNA complexes were prepared at concentrations used for mouse vaccines, taking into account the limited volume that can be administered i.m., i.e. 0.25 µg/µl mRNA. Similar size distributions and aggregation patterns were observed for CAF04:mRNA complexation at a proposed human dose and volume with an mRNA concentration of 0.05 µg/µl (results not shown), indicating the complexation mechanism may be independent of mRNA concentration.

The CAF04:mRNA complexes were also characterized by cryo-TEM, where uncomplexed CAF04 showed the characteristic slightly faceted liposomes, that are observed for DDA-based liposomes [13, 23] (Fig. 1E). Complexation of CAF04 and OVA-mRNA at N/P ratio 1.09 resulted in adsorption of the mRNA on the surface of the liposomal bilayer. The mRNA was indirectly visualized by exposing the sample to the electron beam for 15 s, which caused damage to the surrounding ice due to the higher electron density in the mRNA compared to the water and lipids (Fig. 1E, white arrows). In contrast, at a CAF04:mRNA N/P ratio of 2.18, aggregation of the liposomes was observed in the cryo-TEM pictures. At excess cationic charge, the mRNA may act as a glue causing bridging between liposomes through electrostatic interactions. This was observed by Tanaka et al., where post-insertion of mRNA into LNPs resulted in aggregation and fusion of the LNPs dependent on electrostatic interactions [24]. The cryo-TEM images thus corresponded with the changes in particle size and surface charge observed by DLS at the different CAF04:mRNA N/P ratios.

Protection of CAF04-complexed mRNA from degradation by RNases is a critical parameter. Samples representing complexes with a surplus of mRNA and CAF04, respectively; CAF04:eGFP-mRNA at N/P ratio 1.09 and 2.18 were treated with RNase A and protein kinase K (PK) followed by release of undigested mRNA by a mixture of Triton-X 100 and heparin (Tx-100/hep) (Fig. 1F). The mRNA control was fully digested by RNase A. Furthermore, treatment with Tx-100/hep fully released mRNA from the CAF04:mRNA complexes not incubated with RNase A. Interestingly, a large fraction of the mRNA appeared to be degraded for both formulations following RNase A treatment, though most was degraded at N/P ratio 1.09. However, in the RNase A treated sample, where mRNA was not released by Tx-100/hep, mRNA was detectable in the well, indicating continued complexation with CAF04. This was in contrast to previous studies, where mRNA complexation with cationic nanoparticles resulted in complete RNase protection [25].

The complexation strength of mRNA to CAF04 was evaluated by using heparin displacement on CAF04:eGFP-mRNA at N/P ratio 1.09 and 2.18 (Fig. 1G). In accordance with the gel electrophoresis assay, free mRNA was detected in CAF04:eGFP-mRNA N/P ratio 1.09 at 0 mg/ml heparin, while no free mRNA was detected at N/P ratio 2.18. The levels of eGFP-mRNA released correlated with the concentration of heparin, with CAF04:eGFP-mRNA N/P ratio 1.09 releasing most mRNA at all heparin concentrations, with 100% mRNA released at 4 mg/ml. The release profiles of the two N/P ratios correlated well with the observed structures in the cryo-TEM images, where mRNA was located at the liposome surface at N/P ratio 1.09 indicating easier access for heparin to compete for cationic charges on CAF04. In contrast, mRNA situated between partially aggregated liposomes as observed for N/P ratio 2.18, would prevent heparin access, and thereby hinder mRNA release. 

Next, we investigated how the CAF04:eGFP-mRNA complexes at different N/P ratios affected the transfection efficiency in vitro in THP-1 cells using serum-free cell culture media during the first 4 h of transfection. The CAF04:eGFP-mRNA N/P ratio had a dramatic impact on the expression of eGFP in THP-1 cells, with an N/P ratio of 2.18 resulting in the highest transfection at approx. 40% eGFP^+^ THP-1 cells and corresponding high mean fluorescence intensity (MFI, Fig. 1H, I). Both at higher and lower N/P ratios, the eGFP expression rapidly declined. The eGFP expression was confirmed by imaging at selected N/P ratios by fluorescent microscopy (Fig. 1J). Furthermore, co-incubation with fluorescently labelled DiD-CAF04 showed that the adjuvant adsorbed effectively to the cells at N/P ratios above 2.18, but low levels of DiD^+^ cells were observed at N/P ratio 1.09 (Fig. 1J). This indicates, that the cellular association of CAF04:mRNA to the cells may be influenced by the particle surface charge. Furthermore, the THP-1 cells may preferably take up larger particle aggregates, resulting in the observed optimum at N/P ratio 2.18. The cytotoxicity, measured by flow cytometry with a viability stain of the CAF04:mRNA treated cells, increased with increasing N/P ratios, though the levels of live cells remained above 95% (Suppl. Figure 2 A). However, the number of cells recovered declined significantly in correlation with increasing N/P ratios (Suppl. Figure 2B), indicating that the cells taking up the particles were lysed in response to the increasing cationic charge, while the remaining cells remained viable.

CAF04 can be stored for five years at 4 °C, but also be freeze or spray-dried for extended storage and reducing the need for cold-chain distribution [26, 27]. Therefore, we investigated the transfection efficacy of rehydrated, freeze-dried CAF04 compared to fresh CAF04 following complexation with eGFP-mRNA at N/P ratio 2.18, and found the complexes induced similar transfection levels in THP-1 cells (Suppl. Figure 2 C, D).

We further investigated how the presence of serum proteins (supplied as FCS to the cell culture media) affected the transfection efficacies of CAF04:eGFP-mRNA at N/P ratios 1.09 and 2.18 after 24 h of transfection. The presence of serum proteins reduced the transfection efficacies both as percentage of eGFP^+^ cells and MFI of these cells (Fig. 1K). The reduced transfection efficacy might be due to displacement of mRNA from the CAF04 liposomes, causing both a loss of mRNA and the formation of a protein corona, which may hinder cellular uptake. This was in accordance with the observation that formation of a protein corona on cationic liposome: DNA lipoplexes reduced cellular uptake and transfection efficacy. The formation of a protein corona on the nanoparticles may favor cellular uptake by macropinocytosis over caveolin-mediated endocytosis, which might subsequently affect processing and release of the cargo intracellularly [28]. However, transfection levels were still significant in the presence of serum proteins, indicating the CAF04:mRNA complexes would be active in vivo.

### The ratio of CAF04:mRNA affected the CD8^+^ T-cell response

**Fig. 2:**
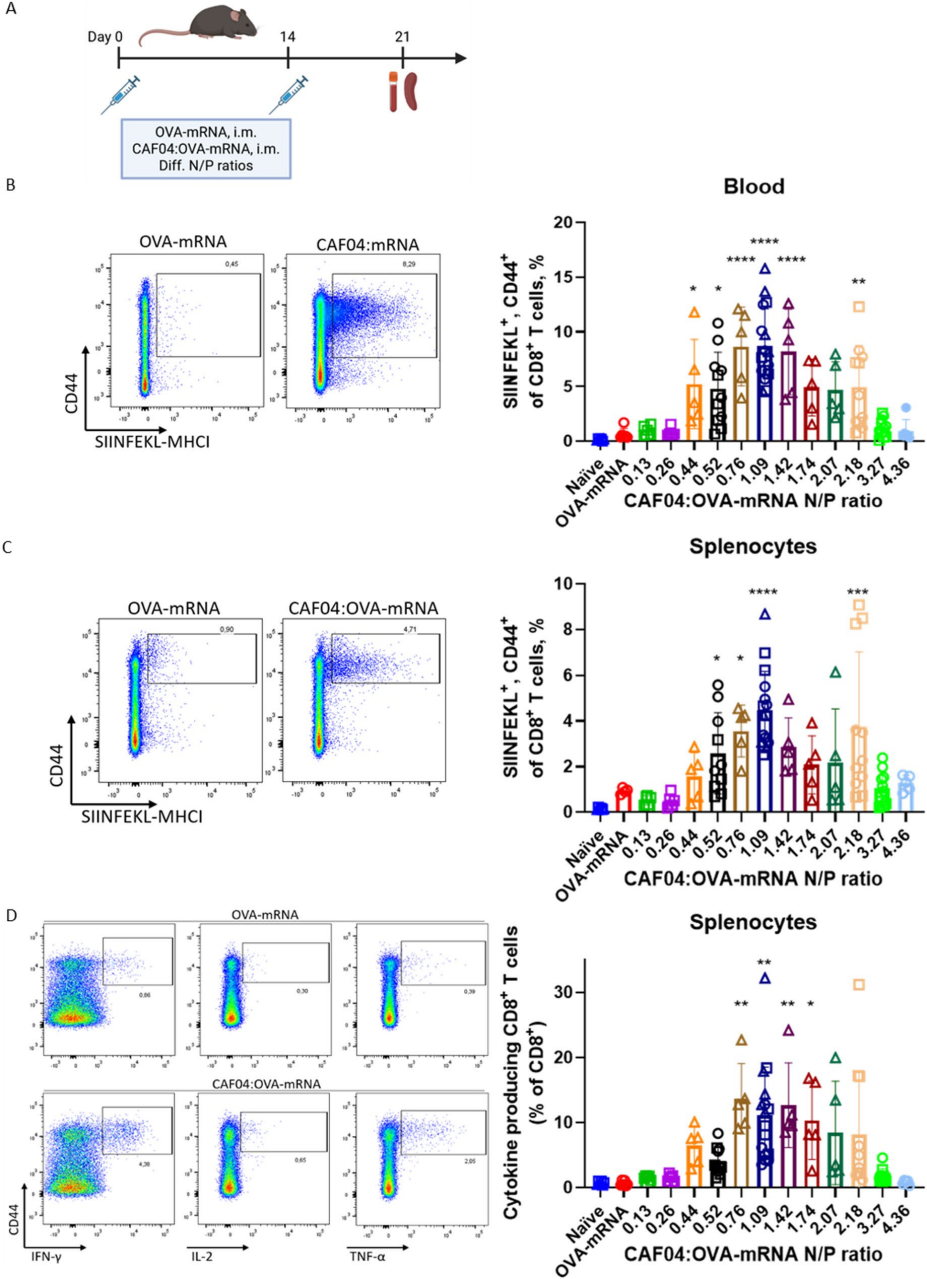
Immunogenicity dependency on CAF04:OVA-mRNA N/P ratio. **(A)** Female C57BL/6 mice were immunized twice i.m. with a two-week interval in alternating quadriceps with 5 µg/dose OVA-mRNA complexed with CAF04 at different N/P ratios. Antigen-specific CD8^+^ T cells were evaluated by staining with fluorescently labelled SIINFEKL:H2-Kb-MHC-I and appropriate antibodies and assayed by flow cytometry in **(B)** the blood and **(C)** splenocytes. **(D)** Splenocytes were stimulated for 6 h with SIINFEKL and IFN-γ, IL-2 and/or TNF-α producing CD8^+^ T cells were assessed by intracellular flow cytometry, which are shown as the level of CD8^+^ T cells producing any cytokine defined by the OR gate in FlowJo. Representative plots of (A, B) SIINFEKL-MHC-I^+^/CD44^+^ and (C) IFN-γ^+^, IL-2^+^ and TNF-α^+^/CD44^+^ CD8^+^ T cells are presented left of the graphs (following the gating strategies in Suppl. Figure 3). Data represents the combined results of three independent experiments (each defined by circles, squares and triangles; not all N/P ratios were included in all experiments, total mice pr. group in plots: Naïve [6], OVA-mRNA [6], N/P ratio 0.13 [5], 0.26 [5], 0.44 [5], 0.52 [11], 0.76 [5], 1.09 [16], 1.42 [5], 1.74 [5], 2.07 [5], 2.18 [11], 3.27 [11], 4.36 [6]). The data represent mean ± S.D., each dot represent an individual mouse. Statistical significance was assessed by one-way ANOVA with Dunnett’s posttest comparing results to the naïve control group, * *p* ≤ 0.05, ** *p* ≤ 0.01, *** *p* ≤ 0.001, **** *p* ≤ 0.0001. A) was created in BioRender: Pedersen, G. (2026) https://BioRender.com/hzkos3e

After showing effective in vitro transfection with CAF04:eGFP-mRNA complexes, we evaluated the immunogenicity of the complexes in vivo in female C57Bl/6 mice, using ovalbumin (OVA) as a model antigen. Results from the in vitro studies in THP-1 cells showed a strong dependence of the CAF04:mRNA N/P ratios on the cellular expression of eGFP, with the optimal ratio at 2.18 (w/w). However, different uptake pathways may be at play in vivo dependent on the targeted cells and therefore, CAF04:OVA-mRNA (5 µg/dose) was administered i.m. at different N/P ratios ranging from 0.13 to 4.36 and CD8^+^ T-cell responses, measured by tetramer staining (SIINFEKL) were evaluated in blood and splenocytes (Fig. 2A-D, Suppl. Figure 3). An optimum in the induced CD8^+^ T-cell responses were observed at CAF04:mRNA N/P ratios 0.76–1.42, corresponding to weight ratios of 1.75–3.25 (Table 1). Thus, there was little correlation between the optimal N/P ratios for eGFP expression in vitro and induction of immune responses in vivo. It appeared that the optimal N/P ratios for in vivo immunizations were when the cationic liposomes were completely coated with mRNA, and therefore had a net negative surface charge. Possibly, this was partly due to the aggregation of the particles observed at high N/P ratios, which is hypothesized to reduce interaction with and uptake by target cells. Furthermore, there was an increased risk of cytotoxicity as the dose of cationic lipids increased. Overall, CAF04:OVA-mRNA N/P ratios above 2.18 resulted in almost complete abrogation of the CD8^+^ T-cell responses and responses were also reduced when the N/P ratio was lower than 0.44, which is possibly because the amount of free mRNA is increasing with fewer cationic binding sites available. Unbound mRNA is rapidly degraded upon immunization, and will thus not contribute to inducing an immune response. In addition, the CAF04 dose was comparably low at lower weight ratios, and might thus not contribute with a sufficient immunostimulatory effect. Confirming the tetramer staining, restimulation with SIINFEKL peptide and analysis of IFN-γ, IL-2 and TNF-α producing cells, showed that optimal responses were found at CAF04:mRNA N/P ratios of 0.76–1.42 (significantly higher than in naïve animals, *p* < 0.01). Overall, CAF04:mRNA N/P ratios of 0.76–1.42 induced high-magnitude CD8^+^ T-cell responses in vivo, with a N/P ratio of 1.09 as the most optimal. We therefore continued using this N/P ratio for further studies.

**Fig. 3:**
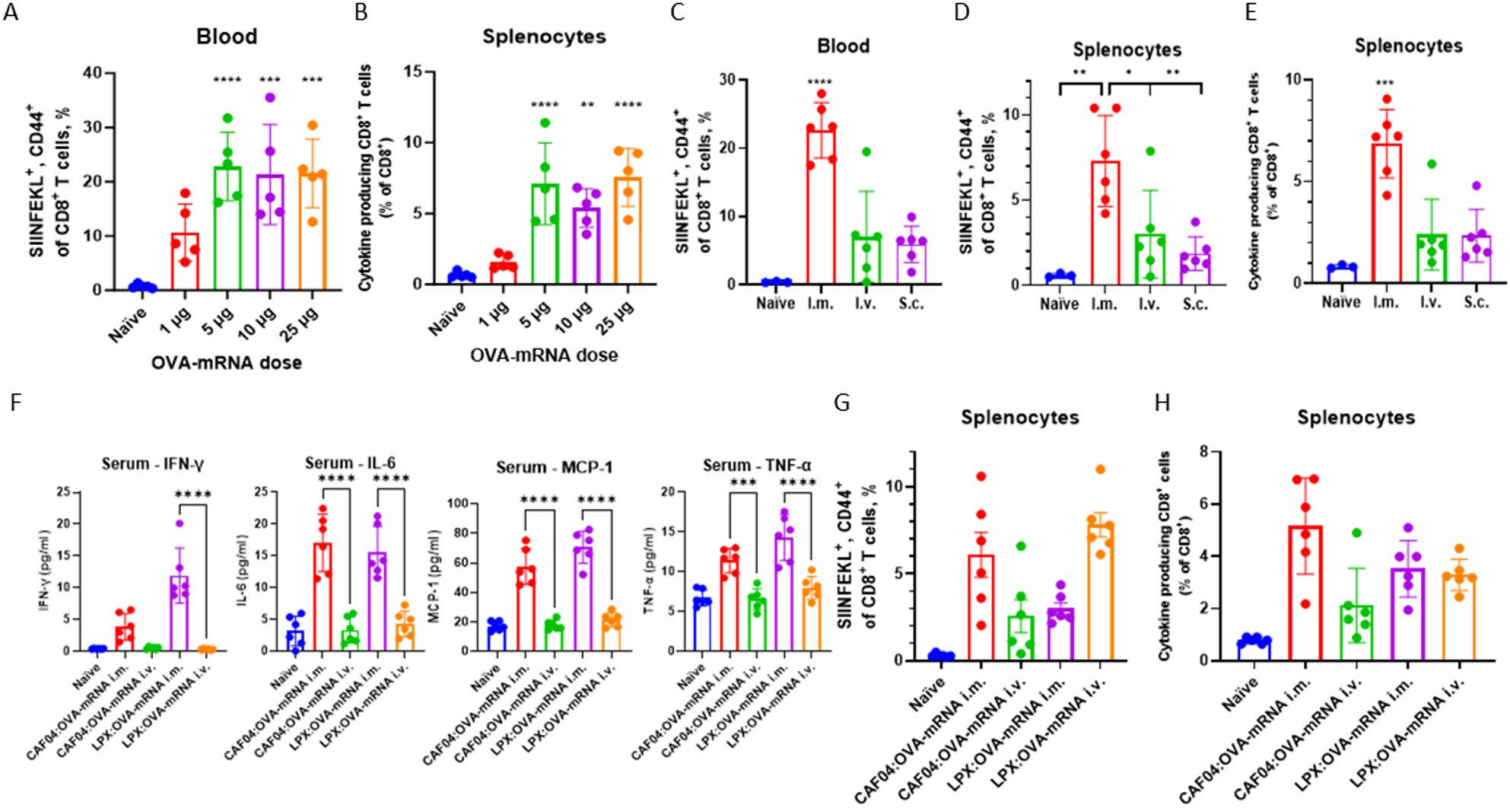
Immune responses depending on OVA-mRNA dose and injection site. Responses to different OVA-mRNA doses were determined by immunizing female C57BL/6 mice twice i.m. with a two-week interval in alternating quadriceps with 1–25 µg/dose OVA-mRNA complexed with CAF04 at an N/P ratio of 1.09. **(A)** Antigen-specific CD8^+^ T cells were evaluated by staining with fluorescently labelled SIINFEKL:H2-K^b^-MHC-I and appropriate antibodies and assayed by flow cytometry in the blood. **(B)** Splenocytes were stimulated for 6 h with SIINFEKL and IFN-γ, IL-2 and/or TNF-α producing CD8^+^ T cells were assessed by intracellular flow cytometry. The antigen-specific CD8^+^ T-cell responses induced following two immunizations two weeks apart i.m. i.v. or s.c. in **(C)** the blood and **(D)** splenocytes were evaluated by staining with fluorescently labelled SIINFEKL:H2-K^b^-MHC-I and appropriate antibodies and assayed by flow cytometry. **(E)** Splenocytes were stimulated for 6 h with SIINFEKL and IFN-γ, IL-2 and/or TNF-α producing CD8^+^ T cells were assessed by intracellular flow cytometry. The CD8^+^ T-cell responses were compared for OVA-mRNA complexed with either CAF04 at N/P ratio 1.09 or LPX (DOTMA:DOPE 1:1 molar ratio) at N/P ratio 0.29 administered twice i.m. or i.v. **(F)** The levels of IFN-γ, IL-6, MCP-1 and TNF-α in serum at 24 h after the 1st immunization assayed by electrochemiluminescence. **(G)** At one week after the final immunization, antigen-specific CD8^+^ T cells in the spleen were evaluated by staining with fluorescently labelled SIINFEKL:H2-K^b^-MHC-I and appropriate antibodies and assayed by flow cytometry. **(H)** Splenocytes were stimulated for 6 h with SIINFEKL and IFN-γ, IL-2 and/or TNF-α producing CD8^+^ T cells were assessed by intracellular flow cytometry. Flow cytometry assays were performed as described in Fig. 2. The data represent mean ± S.D., *n* = 3, 5 or 6 mice/group, each dot represent an individual mouse. Statistical significance was assessed by one-way ANOVA with Tukey’s posttest and asterisks signifies difference to the naïve group unless otherwise indicated, * *p* ≤ 0.05, ** *p* ≤ 0.01, *** *p* ≤ 0.001, **** *p* ≤ 0.0001

The dose of OVA-mRNA was varied to identify the optimal dose inducing the strongest CD8^+^ T-cell responses. Immunization with 5, 10 and 25 µg/dose OVA-mRNA admixed with CAF04 at a 1.09 N/P ratio resulted in similar levels of antigen-specific and cytokine (IFN-γ, IL-2 and TNF-α) producing CD8^+^ T cells in the blood and splenocytes, respectively (Fig. 3A, B). In contrast, immunization with 1 µg/dose OVA-mRNA failed to induce statistically significant CD8^+^ T-cell responses. Thus, 5 µg/dose was deemed sufficient to induce the optimal immune responses, as increasing the OVA-mRNA dose did not appear to enhance the immune responses further.

The CAF04:mRNA complexation ratio might influence optimal induction of CD8^+^ T-cell responses, where resulting particles showing negative surface charges and complexing sufficient amounts of mRNA were optimal. However, part of the reason for this optimum could also be found in the morphology of the CAF04:mRNA complexes, which displayed aggregates with mRNA wedged between the liposomes at N/P ratio 2.18, but single particles with mRNA on the surface at N/P ratio 1.09. Single particles would likely be less cytotoxic and more easily taken up by immune cells at the injection site compared to larger aggregates. Interestingly, higher CAF04 to mRNA ratios were found to be optimal for in vitro transfection of THP-1 cells, where particles with a positive surface charge were much more effective at transfecting the cells than those with a negative surface charge. Hence it appears that in the in vitro model, the ability to directly interact with the cell surface may be governing transfection efficacy, whereas in vivo other mechanisms are more important for induction of CD8^+^ T-cell responses, possibly reduced cytotoxicity, different uptake pathways and uptake by specific cell subsets.

### Immunization with CAF04:OVA-mRNA induced highly cytotoxic CD8^+^ T-cell responses

The DOTMA:DOPE-based (1:1 molar ratio) LPX:mRNA complexes are administered i.v. in both animal models and clinical trials performed by BioNTech [8, 10], indicating that this might be the optimal route of administration for this system. We therefore tested the CD8^+^ T-cell responses to CAF04:OVA-mRNA following either i.m., s.c. or i.v. immunization. Immunization by the i.m. route resulted in significantly higher levels of antigen-specific CD8^+^ T-cell responses in the blood and spleen compared to the i.v. and s.c. administration routes (Fig. 3C, D). In correlation with this, i.m. immunization induced significantly more cytokine-producing CD8^+^ T cells (Fig. 3E). This indicated that i.m. immunization was the optimal administration route for the CAF04:OVA-mRNA complexes.

To investigate distribution of CAF04:mRNA, we formulated the liposomes with DiR and followed fluorescence locally in the i.m. injected muscle and in draining LNs at 6 and 21 h post i.m. immunization (hpi). DiR-CAF04:mRNA complexes 6 and 24 hpi were shown to remain mainly at the injection site within CD45^+^ cells, with very limited detection in the iliac and inguinal LNs (Suppl. Fig. 4 A-D). This was expected, as the particle sizes at approx. 400 nm promotes retention at the injection site and uptake by APCs. Furthermore, localized biodistribution is also observed when CAF is used as adjuvants for protein subunit vaccines [29]. The effect of MMG-1 on the induced immune response was evaluated by titrating MMG-1 from 0 to 100% of the normal dose of CAF04:OVA-mRNA at N/P ratio 1.09 in mice (Suppl. Figure 4E-F). No differences in induced CD8^+^ T-cell responses were observed, suggesting that the main driver of the CD8^+^ T-cell response is the DDA component, although MMG-1 is important for stabilizing the formulation [13]. However, MMG-1 is not a strong ligand for the mouse Mincle, whereas it effectively activates human Mincle [30]. Therefore, MMG-1 may have an immunostimulatory role in other species.

To further understand the immune responses induced by CAF04:OVA-mRNA and LPX:OVA-mRNA, both formulations were administered i.m and i.v. in mice. At 24 h after the first immunization, the levels of proinflammatory cytokines in serum were evaluated (Fig. 3F). Apparently, the administration route was decisive of for the induction of pro-inflammatory cytokines, as elevated levels of IL-6, MCP-1 and TNF-α were observed after i.m. immunization with both formulations, whereas i.v. immunization did not induce any increase in cytokine levels. No induction of IFN-α, IFN-β, IL-1β, IL-5, IL-12p70, or KC/GRO were observed for any of the groups (results not shown). The only difference in the measured cytokines between the formulations was observed for IFN-γ, where LPX: OVA-mRNA induced significant levels, whereas CAF04:OVA-mRNA did not. In contrast, the levels of antigen-specific CD8^+^ T cells in the spleen one week after the final immunization induced by the two formulations were highly dependent on the administration route (Fig. 3G). Thus, it was confirmed that CAF04:OVA-mRNA was most potent following i.m. immunization whereas LPX:OVA-mRNA performed best following i.v. immunization. While this trend was also observed for cytokine-producing CD8^+^ T-cells following CAF04:OVA-mRNA immunization, no difference for this population was observed between i.m. and i.v. immunization with LPX: OVA-mRNA (Fig. 3H).

**Fig. 4:**
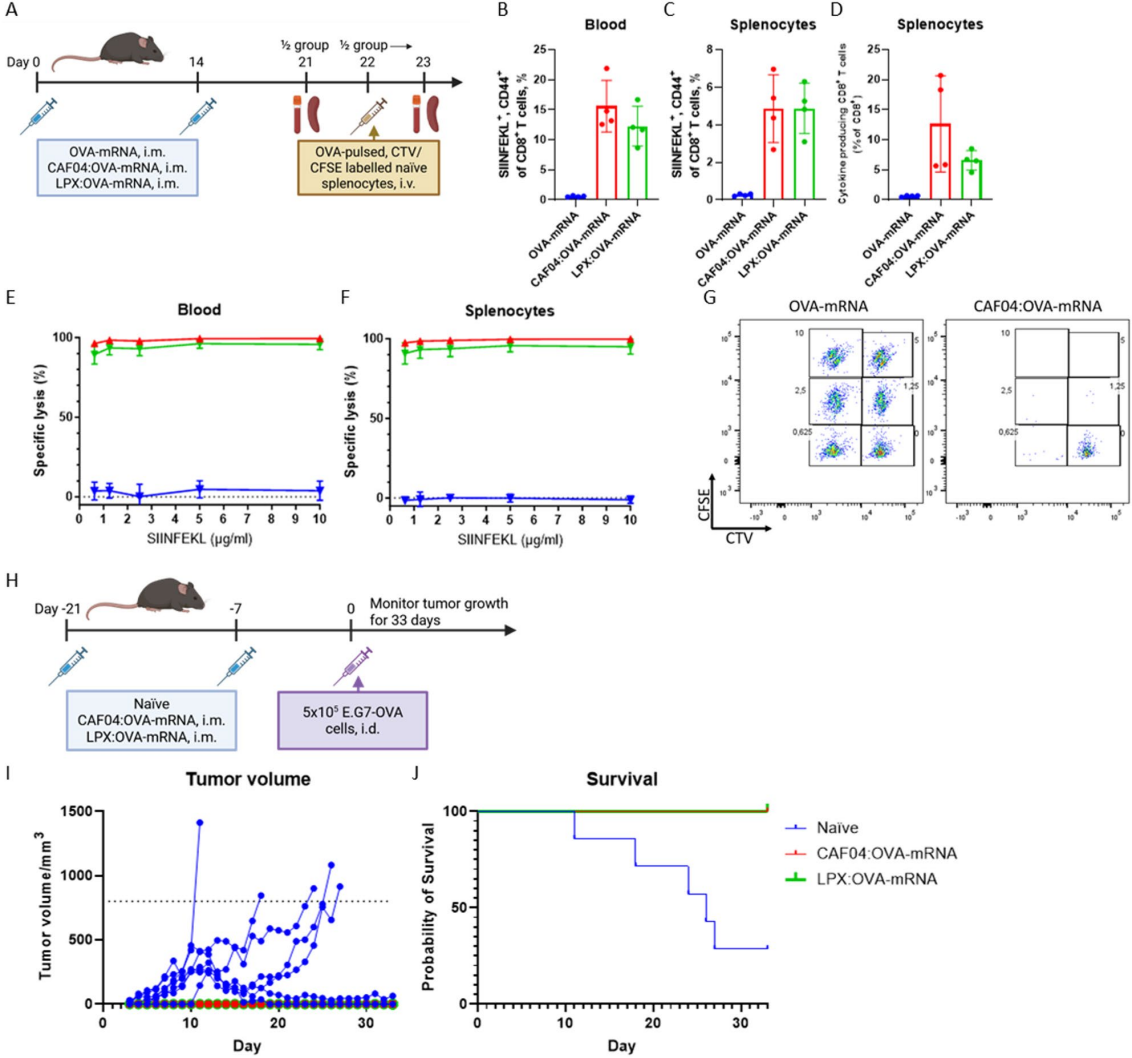
Prevention against tumor growth in a prophylactic tumor model. **(A)** Immunogenicity and cytotoxic potential of CAF04:mRNA complexes (N/P ratio 1.09) were compared with LPX (DOTMA:DOPE 1:1 molar ratio) complexed with OVA-mRNA at N/P ratio 0.29 in female C57BL/6 mice (4 mice/group) immunized twice i.m. with a two-week interval in alternating quadriceps with 5 µg/dose OVA-mRNA. The levels of **(B)** antigen-specific CD8^+^ T cells in the blood and **(C)** spleen and **(D)** cytokine (IFN-γ, IL-2 and/or TNF-α) producing CD8^+^ T cells in the splenocytes were evaluated. Flow cytometry assays were performed as described in Fig. 3. **(E)** The in vivo cytotoxic potential of the antigen-specific CD8^+^ T cells was evaluated by intravenous administration of SIINFEKL-peptide-pulsed splenocytes from naïve mice. Populations of naïve splenocytes were pulsed with different conc. of SIINFEKL (0.0625–10 µg/ml) and stained with different levels of CFSE and CTV, non-pulsed cells were used as a negative control. The mice were euthanized 24 h after, and the levels of peptide-pulsed splenocytes to non-pulsed splenocytes within the mouse were calculated in the blood and **(F)** the spleen. **(G)** Plots of SIINFEKL-pulsed, CFSE and CTV stained splenocytes in the spleen of mice immunized with OVA-mRNA or CAF04:OVA-mRNA, the number for each gate refers to the SIINFEKL pulsing conc. (µg/ml). Bars are mean ± S.D., points are individual mice, *n* = 4. **(H)** Female C57BL/6 mice (7–8 mice/group) were immunized i.m. in alternating quadriceps on day 0 and 14 with 5 µg/dose OVA-mRNA complexed with CAF04 or LPX. On day 21, the mice were inoculated with 5 × 10^5^ E.G7-OVA tumor cells in 50 µl PBS i.d. in the flank. A naïve group was administered Tris-buffer. **(I)** Tumor volume was monitored daily, with a humane endpoint at 800 mm^3^. **(J)** Survival of tumor inoculated mice represented in G). A) was created in BioRender: Pedersen, G. (2026) https://BioRender.com/513p0z1 and H) Pedersen, G. (2026) https://BioRender.com/xwyaljk

The functionality of the CD8^+^ T-cell responses induced by CAF04:OVA-mRNA immunization was compared to LPX:OVA-mRNA complexed at N/P ratio 0.29 as benchmark for i.m. immunization. Female, C57Bl/6 mice were immunized twice with 5 µg OVA-mRNA complexed with CAF04 at N/P ratio 1.09 or LPX at N/P ratio 0.29 (Fig. 4A). Both formulations induced significant CD8^+^ T-cell responses, both at the level of antigen-specific and cytokine producing cells (Fig. 4B-D). CAF04:OVA-mRNA induced similar immune responses as compared to LPX:OVA-mRNA administered i.m., indicating that CAF04 performs on par with the well-established LPX platform. In contrast, no antigen-specific CD8^+^ T cells were induced by naked OVA-mRNA.

The cytotoxic potential of the antigen-specific CD8^+^ T cells was evaluated in vivo by i.v. administering splenocytes from naïve mice pulsed with different concentrations of SIINFEKL (0–10 µg/ml) and distinguished by a combination of CFSE and CTV (Fig. 4E-G). After 24 h, the mice were euthanized and the specific lysis was calculated using the non-pulsed population as control, and relative to a naïve control group of mice. By administering splenocytes pulsed with different SIINFEKL concentrations, it is possible to assess the level of cytotoxicity of the antigen specific CD8^+^ T cells and identify differences between vaccines, which may not be evident if using only one SIINFEKL concentration. Immunization with both CAF04:OVA-mRNA and LPX:OVA-mRNA resulted in potent cytotoxic activity, which effectively lysed the SIINFEKL-pulsed splenocytes at all the tested SIINFEKL concentrations.

### Immunization with CAF04:OVA-mRNA prevented establishment of OVA-expressing E.G7-tumors and delayed growth of established tumors

After the potential of CAF04:OVA-mRNA complexes to elicit cytotoxic T cells was established, the efficacy of these cells was evaluated in a prophylactic cancer model using OVA-expressing E.G7 tumor cells. The mice were immunized twice i.m. on day −21 and −7 with 5 µg OVA-mRNA complexed with CAF04 or LPX in Tris-buffer (10 mM, pH 7.0), with a naïve group only administered Tris-buffer (negative control) (Fig. 4H). The tumor cells were inoculated i.d. at the flank with 5 × 10^5^ cells in 50 µl PBS. Only the naïve group developed tumors, which reached the humane endpoint of 800 mm^3^ for 5/7 mice (Fig. 4I-J). Spontaneous regression has been observed in the E.G7 tumor model, explaining that not all mice in the naïve group reached the humane endpoint in tumor volume [31]. The mice immunized with CAF04:OVA-mRNA (8/8 mice/group) or LPX: OVA-mRNA (7/7 mice/group) were completely protected against establishment of tumors, which illustrates the potential of these adjuvants for cancer vaccines (Fig. 4I-J).

**Fig. 5:**
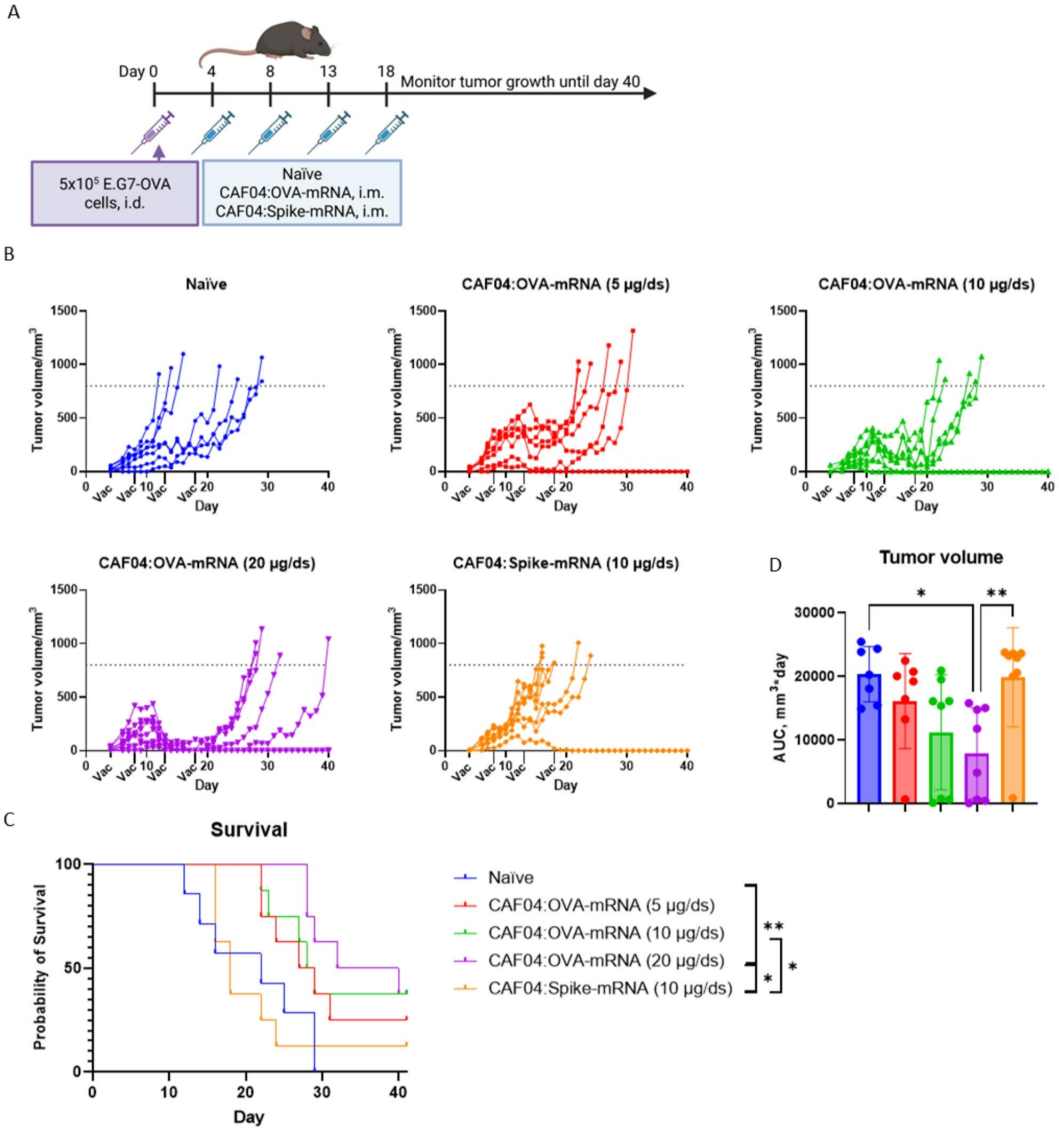
mRNA-dose dependent delay of tumor growth in a therapeutic tumor model. Therapeutic efficacy of CAF04:OVA-mRNA i.m. immunization in an E.G7-OVA s.c. tumor model. **(A)** C57BL/6 mice (7–8 mice/group) were inoculated i.d. in the right flank with 5 × 10^5^ E.G7-OVA cells in 50 µl PBS, followed by immunization four times on day 4, 8, 13 and 18 i.m. in alternating quadriceps with 5, 10 or 20 µg/dose OVA-mRNA complexed with CAF04 at N/P ratio 1.09, with CAF04:Spike-mRNA (10 µg/dose) and Tris-buffer immunized groups as negative control. **(B)** Tumor volume was monitored daily, with a humane endpoint at 800 mm^3^. **(C)** Survival of tumor inoculated mice represented in B). Statistical significance was assessed by Log-rank (Mantel-Cox) test, * *p* ≤ 0.05, ** *p* ≤ 0.01. **(D)** Tumor volume area under curve (AUC). Bars are mean ± S.D., points are individual mice. Statistical significance was assessed by Kruskal-Wallis test followed by Dunn’s posttest, * *p* ≤ 0.05, ** *p* ≤ 0.01. A) was created in BioRender: Created in BioRender. Pedersen, G. (2026) https://BioRender.com/0op7ij7

In a therapeutic cancer study, 5, 10 and 20 µg/dose OVA-mRNA complexed with CAF04 at N/P ratio 1.09 were i.m. administered on day 4, 8, 13 and 18 after inoculation with 5 × 10^5^ E.G7-OVA tumor cells (Fig. 5A). As negative controls, groups of mice were administered CAF04:Spike-mRNA (10 µg/dose) and Tris-buffer. Tumor growth was delayed in a dose dependent manner following immunization with CAF04:OVA-mRNA, with 20 µg/dose OVA-mRNA resulting in significantly increased survival rates (Fig. 5B-C). In contrast, immunization with CAF04:Spike-mRNA did not delay tumor growth. The AUC for the tumors was calculated setting the tumor volume to the humane endpoint of 800 mm^3^ for the remainder of the study, when the mice were euthanized to correct for the incomplete growth curve [22]. The group immunized with CAF04:OVA-mRNA (20 µg/dose) had significantly lower AUC compared to the naïve and CAF04:Spike-mRNA immunized groups (Fig. 5D). Furthermore, there was a trend towards a dose-dependent reduction in tumor volume AUC in the groups immunized with CAF04:OVA-mRNA.

Intradermal (i.d.) immunization with DOTAP:DOPE liposomes complexed with OVA-mRNA protected against subcutaneous E.G7 tumor challenge, and delayed growth of established F10.9-OVA tumors [32]. I.v. immunization with LPX:antigenic-mRNA was effective as both prophylactic and therapeutic vaccines [8]. Another i.v. administered cationic liposomal system complexed with mRNA protected against establishment of E.G7-OVA tumors, but did not significantly hinder growth of established tumors [33]. Our results showing that cationic liposomes can effectively be combined with mRNA to induce CD8^+^ T cells with high cytotoxic potential are in accordance with studies using other cationic liposome platforms, but few studies have evaluated these for i.m. immunization. Hess et al. found that i.d. immunization was not efficient and i.v. administration induced more robust immune responses, while s.c. immunization did not induce any immune responses [32]. Although i.v. administration route for LPX is preferred [8, 10], we found that i.m. immunization induced robust and reproducible CD8^+^ T-cell responses, both when using CAF04 and LPX as delivery system. In contrast, i.v. and s.c. administration of CAF04:mRNA induced lower levels of antigen-specific CD8^+^ T-cell responses. This suggests different mechanisms of action for CAF04 and LPX. It should be noted that we only evaluated LPX:mRNA i.m. for comparison with CAF04:mRNA, and thus its performance after i.v. or s.c. administration in these studies is not known. Also, the LPX:mRNA complexation is performed using a non-disclosed, proprietary protocol [8], which may affect the resulting physicochemical characteristics of the complex and thereby the vaccine efficacy when administered by different routes. Delivering the vaccine by the i.m. route may be advantageous to i.v. immunization as this could retain the vaccine at the site of injection and minimize the risk of systemic cytotoxicity. I.v. immunization poses an increased risk of severe adverse events, e.g. anaphylactic shock, especially with repeated administrations. Although LPX: mRNA complexes have been administered i.v. in several clinical trials and appears to be well tolerated [9, 10], i.m. immunization is less invasive and could be beneficial if similar cytotoxic efficacy can be achieved.

Complexation of CAF04 and mRNA was facilitated by gentle agitation using pipetting, which resulted in particles with N/P-dependent increases in size distributions compared to the uncomplexed CAF04. Pipetting was chosen for complexation to evaluate the feasibility of formulating the CAF04:mRNA complexes without the use of specialized equipment, thus enabling the use of CAF04 with mRNA of choice for bedside mixing in a clinical setting outside designated formulation facilities. However, the limited RNase protection and displacement of mRNA following heparin treatment indicates that, although CAF04:mRNA was highly immunogenic, further optimization of formulation parameters is warranted. Based on the results presented within this paper, CAF04:mRNA is envisioned as suitable for, e.g., personalized cancer vaccines, which will not require significant scale-up of the batch-sizes. Therefore, optimization should focus on establishing reproducible complexation protocols suitable for use within the clinic.

## Conclusion

Herein we show that complexation of mRNA with the cationic liposome CAF04 induces protective CD8^+^ T-cell responses in both prophylactic and therapeutic E.G7 cancer models. The concept of simple pipette mixing enables stockpiling of CAF04 at the vaccination site and combination with tailored mRNA e.g. in a personalized therapeutic cancer vaccine. Furthermore, there is no need for specialized equipment or personnel, easing the use of the vaccines in various environments.

This work presents a comprehensive evaluation of the CAF04:mRNA complexation concept. Further work should investigate the immunogenicity of CAF04:mRNA in other animal models, e.g. pig and non-human primates, to form the basis for advancing to clinical trials.

## Supplementary Information

Supplementary Material 1

## Data Availability

The datasets generated during the current study are available from the corresponding author upon reasonable request.
