# Supplementary material for: A stable two-component cationic liposome platform for mRNA delivery induces CD8+ T-cell responses and protection in a murine lymphoma model
Source: J Nanobiotechnology. 2026 Mar 7;24:350. doi: 10.1186/s12951-026-04234-3 (PMC13081595; doi:10.1186/s12951-026-04234-3)
Supplement: Supplementary file 1 — Supplementary Material 1 [file 12951_2026_4234_MOESM1_ESM.docx]

**Supplementary material for:**

**A stable two-component cationic liposome platform for mRNA delivery induces CD8^+^ T-cell responses and protection in a murine lymphoma model**

Gabriel Kristian Pedersen^a, b^, Reham Sabah Alhakeem^a^, Ahmad Tami^a^, Dennis Christensen^a,c^, Zahra Shabanian^a^, Rune Fledelius Jensen^a^, Katharina Wørzner^a^, Signe Tandrup Schmidt^a, #^

**Supplementary table 1:** Antibody panels used for flow cytometry

Antigen-specific CD8^+^ T cells

| Antibody | Fluorophore | Clone | Dilution factor |
| --- | --- | --- | --- |
| H2-K^b^-SIINFEKL | PE | - | 3.33 |
| CD8 | PerCp-Cy5.5 | 53-6.7 | 600 |
| CD19 | PE-Cy7 | eBio1D3 | 600 |
| CD44 | APC | IM7 | 200 |
| CD4 | APC-eFluor780 | RM4.5 | 600 |

Intracellular stain panel

| Antibody | Fluorophore | Clone | Dilution factor |
| --- | --- | --- | --- |
| CD44 | FITC | IM7 | 600 |
| CD8 | PerCP-Cy5.5 | 53-6.7 | 600 |
| CD4 | APC-eFluor | RM4.5 | 600 |
| TNFα | PE | MP6-XT22 | 200 |
| IFNγ | PE-Cy7 | XMG1.2 | 200 |
| IL2 | APC | JES6-5H4 | 200 |

Suppl. fig. 1:


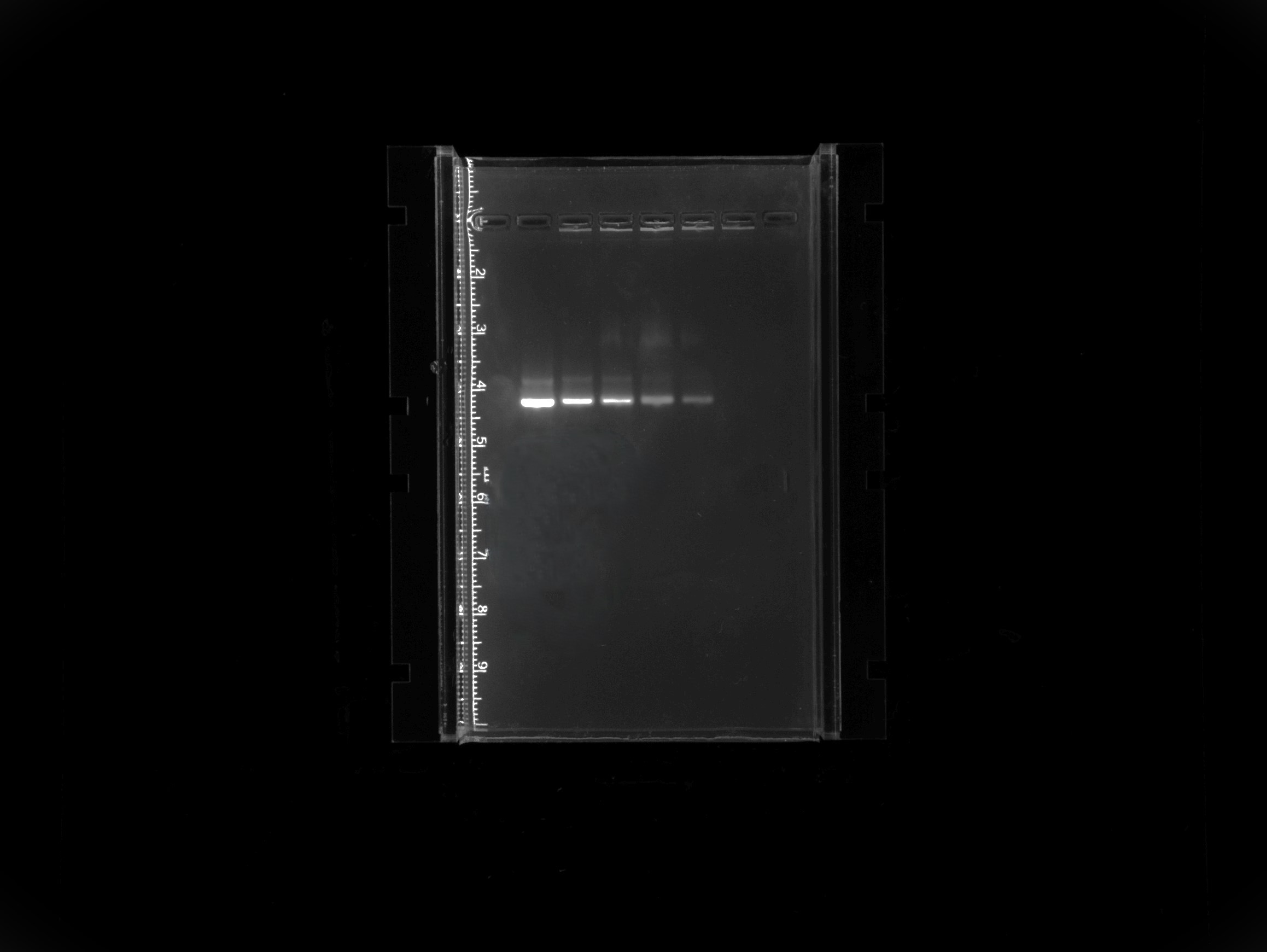


1 2 3 4 5 6

1. eGFP-mRNA
2. CAF04:eGFP-mRNA N/P ratio 1.09
3. CAF04:eGFP-mRNA N/P ratio 1.42
4. CAF04:eGFP-mRNA N/P ratio 1.74
5. CAF04:eGFP-mRNA N/P ratio 2.07
6. CAF04:eGFP-mRNA N/P ratio 2.18

**Suppl. figure 1:** The presence of free mRNA in CAF04:eGFP-mRNA complexed at different N/P ratios were evaluated using a 1% agarose gel with Sybr Green II (ThermoFisher Scientific). Samples were loaded in the lanes as indicated in the figure.

Suppl. fig. 2


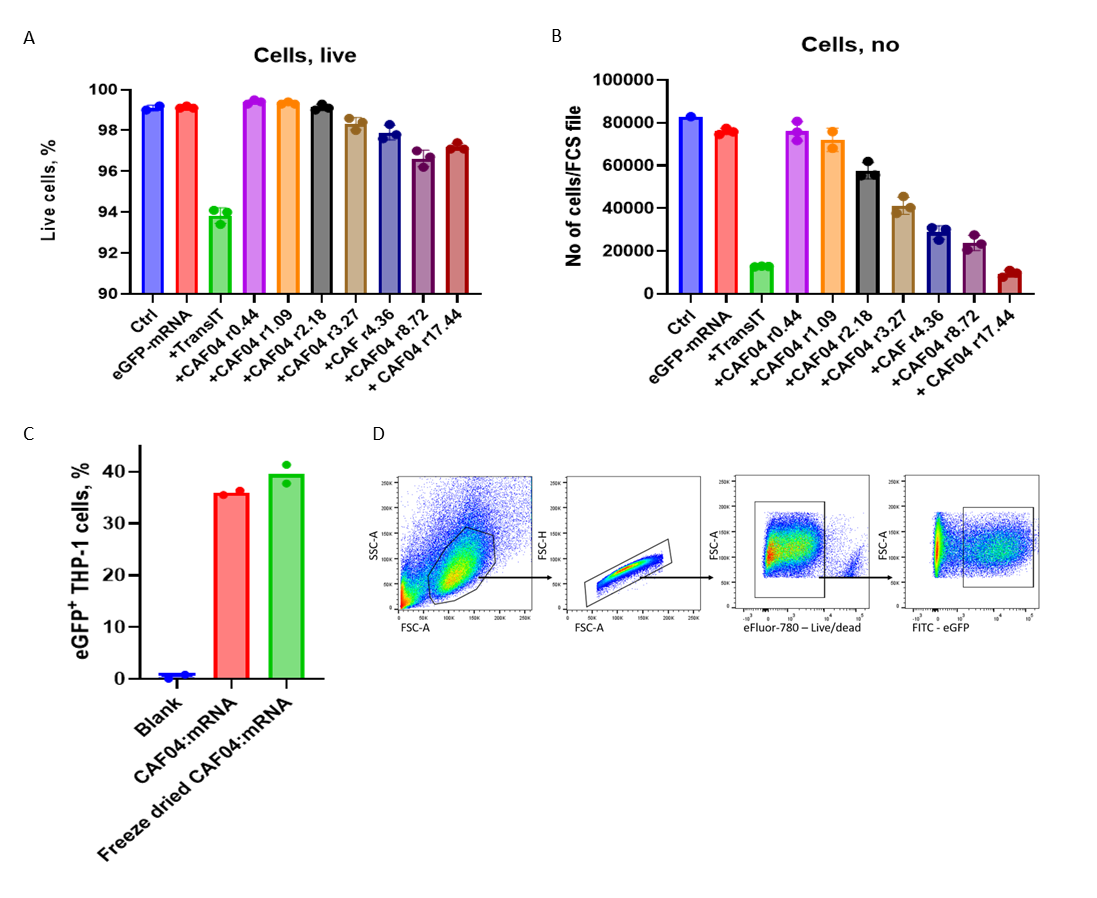


**Suppl. figure 2:** THP-1 cells were stimulated with 0.1 µg eGFP-mRNA/10^5^ cells complexed with different N/P ratios of CAF04 (ratio 0.44-17.44) for 4 h in RPMI. The cells were pelleted by centrifugation and the media changed to cRPMI followed by incubation for 40 h and subsequent analysis of eGFP^+^ cells and live cells by live/dead stain (Fixable viability dye eFluor 780, eBioscience) in flow cytometry. **A)** Live cells determined by flow cytometry live/dead stain and **B)** number of cells in each well determined by flow cytometry. **C)** Freeze-dried CAF04 (using 10% w/v trehalose in Tris-buffer, 10 mM, pH 7.0, as cryoprotectant and freeze dried using a protocol described elsewhere (1)) was rehydrated to the original concentration using mQ water. Freeze-dried, rehydrated and fresh CAF04 was complexed with eGFP-mRNA at N/P ratio 2.18 and used to stimulate THP-1 cells as described above. **D)** Gating strategy for analyzing *in vitro* cells data. n=2-3 technical replicates, the studies were repeated twice.

**References:**

1. Christensen D, Foged C, Rosenkrands I, Nielsen HM, Andersen P, Agger EM. Trehalose preserves DDA/TDB liposomes and their adjuvant effect during freeze-drying. Biochimica et Biophysica Acta (BBA) - Biomembranes. 2007;1768(9):2120-9.

Suppl. fig. 3.


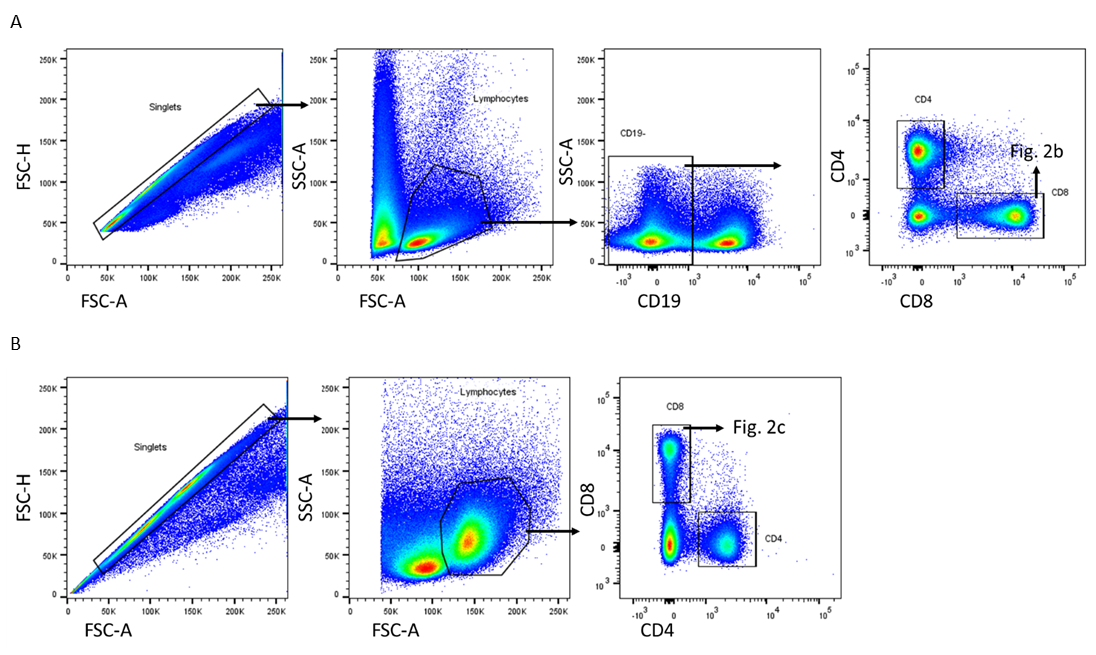


**Suppl. figure 3:** Gating strategies for **A)** H2-K^b^-SIINFEKL FACS and **B)** intracellular FACS, the final gates are depicted in fig. 2b and 2c, respectively.

Suppl. fig. 4


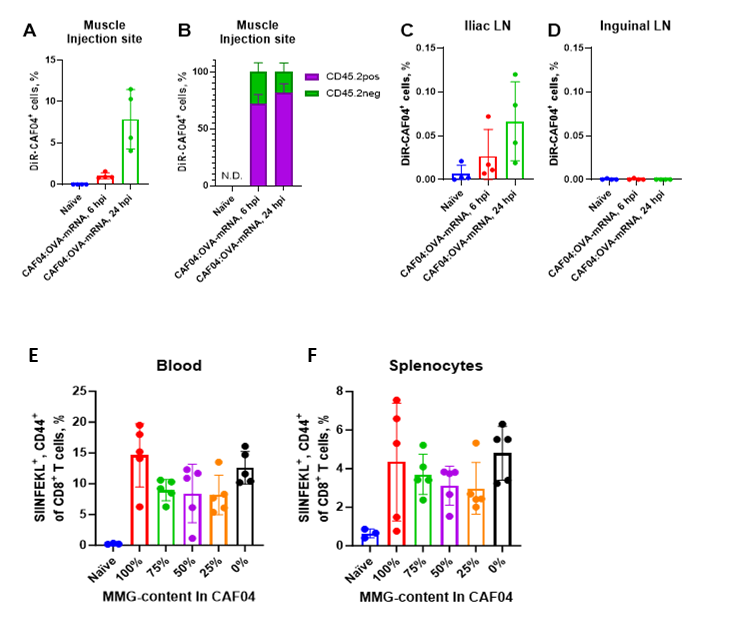


**Suppl. figure 4:** Distribution of fluorescently labelled DiR-CAF04:mRNA (10 µg mRNA/dose, N/P ratio 1.09) following i.m. immunization. C57BL/6 mice were i.m. immunized with DiR-CAF04:mRNA and the distribution in **A)** the muscle, and **C)** iliac and **D)** inguinal lymph nodes were assessed by flow cytometry. **B)** In the muscle, the cells were also stained with anti-CD45.2:PE to assess distribution in hematopoietic versus non- hematopoietic cells. Distribution of the vaccine was evaluated at 6 and 24 hours post immunization (hpi). The effect of MMG-content in CAF04 on the immune responses was evaluated by immunizing female C57BL/6 mice twice i.m. with a two-week interval in alternating quadriceps with 5 µg/dose OVA-mRNA complexed with CAF04 at N/P ratio 1.09. The MMG-content was titrated from 100% to 0% of the normal dose. Antigen-specific CD8+ T cells were evaluated by staining with fluorescently labelled SIINFEKL:H2-Kb-MHC-I and appropriate antibodies and assayed by flow cytometry in **E)** the blood and **F)** splenocytes.
